# Phylogenetics in space: How continuous spatial structure impacts tree inference

**DOI:** 10.1016/j.ympev.2022.107505

**Published:** 2022-05-14

**Authors:** Zachary B. Hancock, Emma S. Lehmberg, Heath Blackmon

**Affiliations:** a Department of Biology at Texas A&M University, College Station, TX 77840, United States; b Ecology & Evolutionary Biology IDP at Texas A&M University, College Station, TX 77840, United States; c Department of Integrative Biology, Michigan State University, 293 Farm Ln., East Lansing, MI 48825, United States

**Keywords:** Gene tree, Hybridization, Multispecies coalescent, Species delimitation, Species tree

## Abstract

The tendency to discretize biology permeates taxonomy and systematics, leading to models that simplify the often continuous nature of populations. Even when the assumption of panmixia is relaxed, most models still assume some degree of discrete structure. The multispecies coalescent has emerged as a powerful model in phylogenetics, but in its common implementation is entirely space-independent – what we call the “missing *z*-axis”. In this article, we review the many lines of evidence for how continuous spatial structure can impact phylogenetic inference. We illustrate and expand on these by using complex continuous-space demographic models that include distinct modes of speciation. We find that the impact of spatial structure permeates all aspects of phylogenetic inference, including gene tree stoichiometry, topological and branch-length variance, network estimation, and species delimitation. We conclude by utilizing our results to suggest how researchers can identify spatial structure in phylogenetic datasets.

## 1. Introduction

Humans have an innate tendency to discretize nature. Taxonomy epitomizes this instinct: it searches for distinct characters, markers, or behaviors that differentiate organisms. Binomial nomenclature, the dichotomous key, all characterizes our desire to categorize biology. These categorizations make superficial sense; a cursory examination of biological diversity appears to support distinct species boundaries (Coyne and Orr 2004). Dawkins and Wong (2016) referred to this human urge as the *tyranny of the discontinuous mind* – a mental state of incongruity at the continuous nature of biology. As alluded to above, an example of this is the “speciation continuum”, which has received increased attention in recent years (e.g., Etienne and Rosindell 2012; Li et al. 2017). For example, Etienne et al. (2014) introduced the “protracted speciation model” that incorporates probabilities of lineage splitting, extinction, merging, and completion. Li et al. (2017) use the protracted speciation model to demonstrate how the microevolutionary processes of lineage extinction and splitting can drive macroevolutionary patterns of species diversity along latitudinal gradients.

In population genetics, most models assume some level of discretization out of mathematical convenience. Discrete population models have no spatial component and are composed of randomly mating individuals. These discrete populations are the foundation of the classical Wright-Fisher (Wright 1931; Fisher 1923) and Moran (Moran 1958) models. Even when models subdivide the population into a series of demes, such as in the stepping-stone model (Kimura and Weiss 1964), each deme still consists of randomly mating individuals. Once a migrant finds their way into a deme, regardless of their deme of origin, they have the same probability of finding a mate as any other deme constituent.

**Fig. 1.**
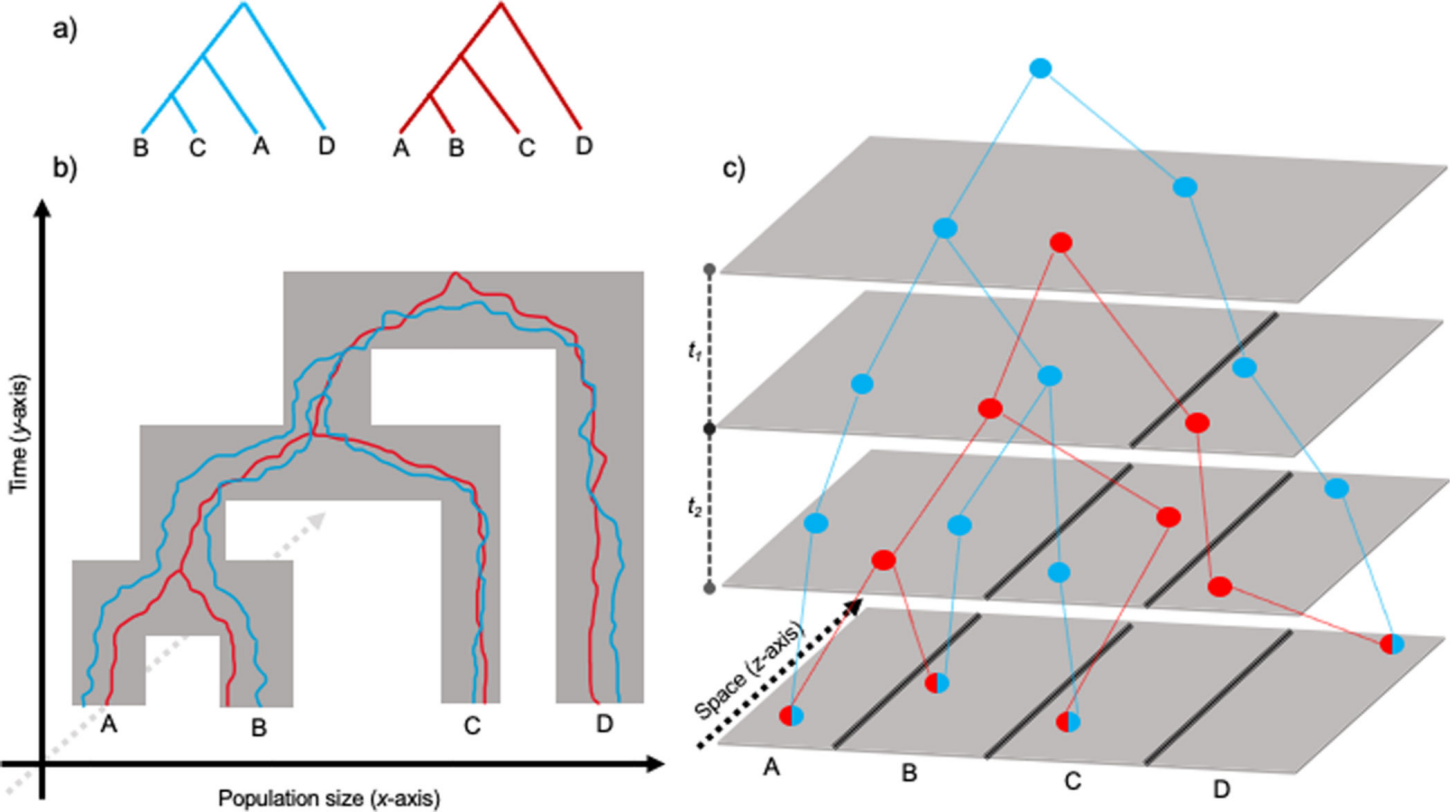
How space can make sense of gene tree discordance. A) Two gene trees, where red matches the species tree and blue is incongruent. B) Gene trees imbedded within the species tree, with time on the y-axis and population size on the x-axis (displayed as the width of the species tree). C) Introducing the spatial component of gene trees; each point is an individual location, with half-color points representing individuals in the present day and solid-colored are their ancestors. Black lines represent barriers to gene flow. Note that despite the barrier between A and B being removed prior to that between B and C, the ancestral locations are quite geographically distant. Therefore, B and C for the blue lineage, due to geographic proximity, coalesce sooner than A and B. In this way, spatial proximity of ancestors is a greater predictor of coalescent times than the species tree. (For interpretation of the references to color in this figure legend, the reader is referred to the web version of this article.)

Phylogenetic inference using the multispecies coalescent model likewise assumes individuals exist independent of space. There are only two dimensions to the model: the width, dictated by the effective size of the population ($N_{e}$), and the length, the temporal aspect (Fig. 1b). Bifurcations in the tree implicitly assume that the descendant $N_{e}$ is immediately in equilibrium and can be represented by a single value (N, τ, or θ). In reality, $N_{e}$ progresses through a period of nonequilibrium the duration of which is dictated by the degree of structure present in the ancestral population (Poissant et al. 2005; Pearse et al. 2006). Furthermore, these divergence events are assumed to occur evenly – lineages are equally likely to end up in either of the two descendant species.

The space-independence or “missing *z*-axis” typical of the models described contribute to their poor performance when populations are actually continuous (Fig. 1; Bradburd and Ralph 2019). Battey et al. (2020) found that many summary statistics (such as ${\text{θ}}_{\text{π}}$, Tajima’s D, and $F_{IS}$) behaved significantly differently in continuous populations versus a random mating population of equal size. While some of the trends were captured in stepping-stone models, strange artifacts emerged; for example, observed heterozygosity and Tajima’s D were far higher at low neighborhood sizes in the stepping-stone models ($\text{neighborhood size}=4\text{πρ}{\text{σ}}^{2}$, where π is the mathematical constant, ρ is the population density, and σ is the dispersal distance; Wright 1943). These may be a feature of both discretizing the habitat and the sample size approaching the local $N_{e}$ when deme size is small (Battey et al. 2020). Wright (1943) noted that for continuous populations to be indistinguishable from panmixia, neighborhood sizes must exceed 1000. This neighborhood size is likely rarely achieved in nature. For example, Jasper et al. (2019) estimated the neighborhood size of the mosquito *Anopheles aegypti* to be only 268. Clearly, space matters for the inference of population genetic data. But what about phylogenetics?

Phylogenetic inference rarely incorporates spatial structure as an explanation for gene tree discordance or node height variance. Many phylogeographic studies incorporate population genetic analyses, including analyzing population structure, but this tends to be a separate analysis that doesn’t directly influence the phylogenetic interpretation (e.g., Waldrop et al. 2016; Domingos et al. 2017; Takada et al. 2018; but see Lemey et al. 2010). Instead, most of the variance in tree topology is attributed to incomplete lineage sorting (ILS) or hybridization (Cui et al. 2013; Árnason and Halldórsdóttir 2019; MacGuigan and Neer 2019) and branch-length variation to the action of natural selection or substitution rate heterogeneity (which may be influenced by population structure, though this is rarely directly invoked; Bromham et al. 2017). This is not due to a lack of theoretical or empirical work that demonstrates the importance of spatial structure (e.g., Wakeley 2000; Slatkin and Pollack 2009; DeGiorgio and Rosenberg 2016). In phylogenetics, the issue may instead be a kind of *tyranny of discontinuous timescales* – that is, the mental state that posits the processes of microevolution act on such different timescales that their influence on macroevolution should be negligible.

Most simulation studies on ILS or species tree inference begin by simulating a species tree under either a pure birth model or a birth-–death model, followed by coalescent simulation of gene trees (e.g., Maddison and Knowles 2006; Leaché and Rannala 2011; Fujisawa et al. 2016). As mentioned above, this method assumes panmictic ancestral populations, and therefore all genetic lineages have equal probability of being split into descendent species (i.e., lineages exist independent of space). This space-independence simplifies ranges by failing to capture how realistic landscape dynamics can shape lineage diversity (Bradburd and Ralph 2019). For example, Wilkins and Wakeley (2002) found that coalescent times in a continuous space population depended not only on the distance between samples, but also the distance the samples were from the range center. Lineages within the range center coalesced deeper in time than peripheral lineages separated by the same distance. This feature of ranges – higher diversity in the center relative to the periphery – is not merely a feature of population genetic models but has been discussed for decades in the rangeland and conservation biology literature (e.g., Hengeveld and Haeck 1982; Lomilino and Channell 1995; Donald and Greenwood 2008).

The specific mode of speciation may contribute to gene tree asymmetry and node height variance as well. Allopatric speciation involves a split following a range expansion into a previously uninhabited territory or some form of range fragmentation. Coyne and Orr (2004) define these two modes of allopatry, originally identified by Mayr (1954), as *peripatric speciation* and *vicariant speciation*, but note that their main difference is in the size of the resulting populations. Each of these modes impact gene trees differently. For example, peripatric speciation is biased towards lineages present in the periphery of the population. Since these lineages tend to be less diverse, this may intensify the founder effect as the population expands into a new territory, especially if dispersal is low (Coyne and Orr 2004; Malay and Paulay 2010; Castellanos-Morales et al. 2016). In a model of vicariant speciation, an initially large population characterized by patterns of isolation-by-distance (IBD) is cut-off from its neighbors. This may lead to species with ancestors historically on opposite ends of the range to be more deeply diverged than expected in a panmictic model (Hancock and Blackmon 2020).

We contend that discounting the spatial component of populations (the *z*-axis; Fig. 1b) can lead to spurious conclusions about both the underlying relationships of species (topology) and their history of divergence (node heights). In this study, we review several key findings in the literature and use continuous population models to illustrate the importance of spatial structure in three aspects of phylogenetics: 1) gene tree asymmetry and inference of hybridization; 2) species tree inference and divergence estimation; and 3) species delimitation. Finally, we propose how these findings can aid in the identification of spatial structure in a dataset, which, we hope, will promote a more thorough investigation of phylogenetics in an explicitly spatial context.

## 2. Trees in space: model

To illustrate the impacts of spatial structure on phylogenetic inference, we constructed a series of continuous-space models for 3-, 4-, and 6-taxon species trees using the forward-time simulator SLiM v3.3 (Haller and Messer 2019). Habitats were modelled as a grid with dimensionality (*x*, *y*), and were subset by how speciation (or population divergence) occurred: 1) vicariant speciation or 2) peripatric speciation (Coyne and Orr 2004). Vicariant speciation begins with a population distributed across the entire range, and proceeds through serial fragmentation events (Fig. S1a). Peripatric speciation, alternatively, begins with a single population that expands into a new territory before being cut-off from its source population (Fig. S1b). Due to the nature of population divergence, population size per population fluctuates, either progressing through a series of expansions or contractions. All simulated species trees are asymmetric or comb-like: ((AB)C), (((AB)C)D), and (((((AB)C)D)E)F).

Population size is an emergent property of the local population density, which is governed by the spatial interaction distance, ${\text{σ}}_{\text{I}}$. The strength of spatial competition and mate choice is drawn from a Gaussian distribution with a maximum of $1/\left[\begin{array}{c} 2\text{π}\;{\text{σ}}_{I}^{2} \end{array}\right]$, where π is the mathematical constant, and is experienced within a maximum distance of $3{\text{σ}}_{\text{I}}$. After mates are chosen, the number of offspring produced are drawn from a Poisson distribution with a rate of 2, which on average replaces the parents. Dispersal occurs immediately after offspring generation. Dispersal distance is drawn from a uniform random distribution with a minimum and maximum of −$3{\text{σ}}_{\text{D}}$ and $3{\text{σ}}_{\text{D}}$, respectively. Following dispersal, the parental generation dies. Simulated individuals in all models are diploid with 1000 Mb haploid genomes with a recombination rate of 10^−9^. Tree-sequences produced from each simulation are uploaded into *tskit* via *pyslim* (Kelleher et al. 2018). We then use the recapitation method in *pyslim* to simulate a coalescent history across all genomic intervals in which there exists multiple roots (i.e., coalescence did not occur during the run; Haller et al. 2019). Using *msprime* (Kelleher et al. 2016), we overlay neutral mutations onto the trees at a rate of 10^−8^. Further details on each analysis performed is presented in the section in which it appears, and all SLiM recipes can be found at https://github.com/zachbhancock/phylo_in_space. Detailed discussion of the model can be found in the Supplementary Materials.

## 3. Slatkin’s skew and gene tree asymmetry

The multispecies coalescent provides an intuitive way to investigate topological incongruence between gene trees. The standard MSC assumes that all gene tree incongruences are the result of ILS, and the pervasiveness of ILS can be predicted given we know something about the intervals between speciation events. This interval is measured in units of $2N_{e}$ (or “coalescent units”). For gene trees that are discordant with the species tree, the presence of each topology is related to the length of intervals between speciation events ($t_{1}$ and $t_{2}$ in Fig. 1). For a species tree (((AB)C)D), focusing only on asymmetric trees, those with topology (((AC)B)D) and ((CB)A)D) result from the lack of coalescence between lineage A and B during the interval *t*_*2*_, and therefore should occur with equal probability (Rosenberg 2002). The same is true for any tree in which D is an ingroup – they can only result from the lack of coalescence during interval $t_{1}$, and therefore all have equal probability. The tree (((AB)D)C) occurs with slightly higher probability because it only relies on lineages (AB) and C not coalescing during the single interval $t_{2}$, as opposed to all three lineages (A, B, and C) not coalescing during either interval $t_{1}$ and $t_{2}$.

These underlying gene tree stoichiometries are the basis for many common tests of hybridization, such as the ABBA-BABA test (Green et al. 2010; Durand et al. 2011) and forms the basis for network approaches to resolving gene tree conflict (Solís-Lemus and Ané 2016). The logic is straightforward; for example, in the case of a 3-taxon tree, when there is a significantly greater proportion of ((BC)A) topologies compared to ((AC)B) topologies, one might infer that gene flow has occurred at some point in the past between lineages B and C.

Slatkin and Pollack (2009) were the first to point out that gene tree asymmetry may not be the result of hybridization, but instead can emerge due to underlying population structure. They imagined a simple 3-taxon tree in which there was a barrier to gene flow in the ancestral lineage of species A and species B + C. They found that the stronger the barrier to gene flow, the greater the gene tree asymmetry became. We refer to this asymmetry due to ancestral population structure as “Slatkin’s skew”.

To illustrate Slatkin’s skew, we simulated 3- and 4-taxon trees in continuous space. These populations were modelled as “clustered” or “unclustered”. Clustered models had low ${\text{σ}}_{\text{D}}$ and ${\text{σ}}_{\text{M}}$, but high ${\text{σ}}_{\text{I}}$ – this resulted in clumping patterns of ancestry across the range. The unclustered model was the opposite – high ${\text{σ}}_{D}$ and ${\text{σ}}_{\text{M}}$ with low ${\text{σ}}_{I}$ – and was meant to approximate a random mating population. For these simulations, we used both the vicariant and peripatric speciation modes to demonstrate the differential impact on gene tree asymmetry. Gene trees were parsed in *tskit* and imported into the R platform (R Core Team 2020) where we used the package *evobiR* (Blackmon and Adams 2015) to count the different topologies among the trees.

**Fig. 2.**
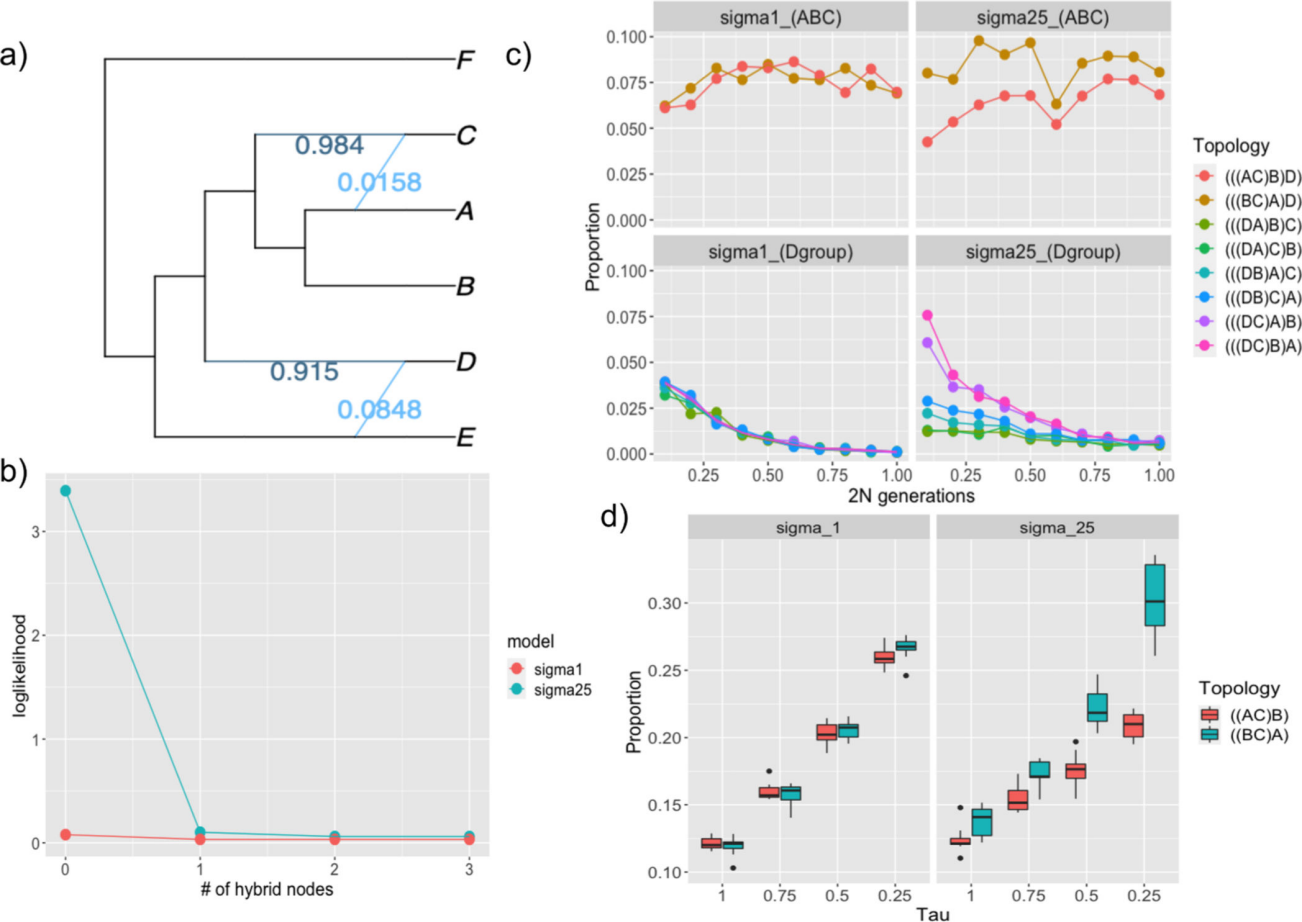
Demonstrating Slatkin’s skew. A) Inferred hybridization using SNaQ; dark blue is the CF for the bifurcating tree and light blue is the discordant proportion; B) change in pseudolikelihood at increasing number of hybrid nodes; C) 4-taxon gene tree distributions of discordant topologies; “sigma1” and “sigma25” are the unclustered and clustered models, respectively. The top row (ABC) represent the distribution of trees where the discordance is either (AC)B or (BC)A, whereas the bottom row is any discordance in which D is an ingroup instead of the outgroup. All of these topologies under the MSC are expected to occur with equal frequency. The x-axis represents the number of generations separating each split. D) 3-taxon tree distributions of discordant topologies. As in (C), these discordant topologies are expected to occur with equal frequency. Note that in all cases of discordance, trees with upwardly-biased distributions are those with taxa geographically closer (e.g., D-C are closer to one another than D-B or D-A in (B), and B-C are closer than C-A in (D)). (For interpretation of the references to color in this figure legend, the reader is referred to the web version of this article.)

Results from these simulations are shown in Fig. 2. For the case of the 3-taxon tree previously investigated by Slatkin and Pollack (2009), we show that the same gene tree asymmetry will arise in continuous-space given that the mode of speciation is vicariance (Fig. 2d). The asymmetry becomes more skewed by geography as *t* approaches 0. As expected, similar trends emerge with the 4-taxon tree: when D is an ingroup, the skew becomes more dramatic as $t_{1}$ and $t_{2}$ in units of 2 *N* fall below 0.75. Interestingly, for the (ABC) coalescence, topology (((BC)A)D) occurs with greater frequency at *all* values of $t_{2}$ in the clustered models, though the difference becomes more extreme at lower $t_{1}$ values (Fig. 2c). When the mode of speciation is peripatry, no skew occurs, and we find that the proportion of ((AB)C) trees are much higher at all t than expected under a neutral MSC model.

Slatkin’s skew has mostly been investigated in the context of human population genetics where there is interest in distinguishing ancestral structure from introgression with Neanderthals (e.g., Krings et al. 1997; Nordborg 1998; Wall 2000; Yang et al. 2012; Schumer et al. 2018). Eriksson and Manica (2012), for example, extended Slatkin and Pollack’s (2009) model to an ancestral stepping-stone and found that the polymorphisms shared between Eurasians and Neanderthals was compatible with a scenario in which no hybridization had occurred. Theunert and Slatkin (2017) replicated their results and proposed using the unconditional site frequency spectrum as a tool to distinguish ancestral population structure from hybridization. However, outside of human population genetics there are few examples in which the impact of ancestral structure is even mentioned as a potential cause of gene tree discordance (but see Degnan 2018; Huynh et al. 2019).

Phylogenetic networks have become a popular tool to visualize gene tree incongruence. Whether the network is implicit (SplitsTree; Huson 1998) or explicit (SNaQ; Solís-Lemus et al. 2017), reticulations are interpreted as evidence for historic gene flow (Degnan 2018). Phylogenomic studies often leverage species tree inference programs to generate a guide-tree for network inference (Cui et al. 2013; Árnason and Halldórsdóttir 2019; MacGuigan and Neer 2019). However, these approaches are not immune from Slatkin’s skew as they are based on the same standard MSC model as the D-statistic.

Using the true gene trees from the 6-taxon simulation as input, we inferred phylogenetic networks using SNaQ with the true species tree as the guide. We performed 10 runs per model, each with the maximum number of hybrid nodes ranging from 0 to 3. Following recommendations from Solís-Lemus et al. (2017), we chose the best number of hybrid nodes based on a slope heuristic (Fig. 2b). For the clustered model, incorporating even a single reticulation improved the pseudolikelihood from 3.392177 to 0.1021881 (Fig. 2b). The improvement for the unclustered model was far less dramatic: no hybrid nodes (loglik=0.0788371) compared to 1 hybrid node (loglik=0.0332104). The unclustered runs never inferred more than 1 hybrid node, regardless of how high the max number was set.

When will Slatkin’s skew matter? In short, when spatial autocorrelation persists between speciation intervals. We can prime our expectations by considering a “separation of timescales” approach to the coalescent (Wakeley, 1999; Wilkins, 2004). The first timescale – the *scattering phase* – is characterized by spatial autocorrelation. Rates of coalescence in this phase are greater than the expected $1/N_{e}$ since lineages nearer one another share an ancestor more recently than expected given the size of the population. The spread of ancestry across a range is diffusive and occurs at a rate of $\text{σ}\sqrt{n}$, where n is the generation and σ is the dispersal distance (Bradburd and Ralph, 2019; Kelleher et al. 2016). Once a lineage has ancestors that have diffused across the entire landscape backward in time, the coalescent enters the *collecting phase* (Wakeley 1999). This phase can be thought of as behaving according to the standard coalescent model, in which the average time to a common ancestor for k=2 is $2N_{e}$ and for k=i is $4N_{e}/[i(i-1)]$. Since the rate of diffusion is dictated by σ, the timescale separating these two phases are proportional to the dispersal potential and the length of the range. If the length of the speciation interval is short relative to the timescale separating the scattering and collecting phases, then the spatial distribution of the ancestral population will dictate gene tree stoichiometry and Slatkin’s skew will be in play. However, for speciation intervals $\geq 2N_{e}$ (or ~ 1.0 coalescent unit), even when dispersal is very low, gene tree proportions are indistinguishable from expectations under the MSC.

## 4. Wicked Forests: Species tree inference and gene tree variance

At a fundamental level, phylogenetic inference seeks to infer the relationships between species (topology) and the distance between them (branch-lengths). The currency of phylogenetics are gene trees, which are themselves biased subsets of the true population genealogy (Kelleher et al. 2016). From the distribution of gene trees, the multispecies coalescent model can be used to infer the species tree – the “true” history of the population – by conditioning a proposed species tree on a set of gene trees (Maddison 1997; Degnan and Rosenberg 2009; Chifman and Kubatko 2014). It’s important to note that while gene trees are real things – whether or not we can observe them directly – species trees are not. They are merely representations of the “true” history of a species in respect to how it has shaped the underlying gene tree distributions.

The genetic distance between any two species is on the order of $T_{D}+2N_{e}$ generations, where $T_{D}$ is the time of divergence (the “true” history). This is because two lineages cannot coalesce until at least time $T_{D}$, and once they are in the same population it on average takes $2N_{e}$ generations for them to coalesce (Wakeley 2000). Since we cannot observe gene trees directly and must rely on mutation to supply us with the information to infer them, it’s useful to note that, assuming panmixia, the pairwise sequence divergence between species 1 and 2 is ${\text{π}}_{12}=2\text{μ}(T_{D}+2N_{e})$, where μ is the per generation mutation rate (Wakeley 2000).

Two important sources of bias from this expectation emerges when the z-axis is considered. First, population structure acts to inflate $N_{e}$ relative to $T_{D}$, which leads to a greater contribution to ${\text{π}}_{12}$ from $2N_{e}$ (Slatkin 1991; Wakeley 2000; Hancock and Blackmon 2020). Second, spatial structure increases the variance in the $T_{MRCA}$ as geographically close lineages coalesce rapidly whereas more distant lineages take much longer (Wall 2000). This second component also impacts the topological concordance of a given gene tree and is biased towards discordant topologies. This is because there is a hard lower-bound to the node height – it cannot be lower than $T_{D}$ – whereas there is no such upper-bound. Therefore, spatial structure is expected to on average inflate branch-lengths and increase topological discordance among gene trees.

In general, we are interested in extracting some estimate of time from node heights. Under neutrality, if we have a good estimate of the per generation mutation rate, μ, then we can estimate time directly as ${\text{π}}_{12}/2\text{μ}$. This is because the rates of substitution and mutation are equivalent under neutrality, and, importantly, independent of $N_{e}$ (Kimura 1987). This finding is generally robust, even in the presence of population structure, as long as the rate of coalescence is still $1/2N_{e}$ (Lehman 2014). However, when $N_{e}$ varies across a landscape, as is often the case in spatially structured populations (Wilkins and Wakeley 2002; Bradburd and Ralph 2019), the rate fluctuates across the range causing the local $N_{e}$ to impact substitution rates (Allen et al. 2015). This has implications for many widely used molecular clocks (e.g., Knowlton and Weigt 1998; Weir and Schluter 2008; Herman et al. 2018), because the calibration source is unlikely to adequately represent the spatial population dynamics of a different species. However, methods that don’t assume a strict clock, such as several relaxed clocks methods (Drummond et al 2006; Lepage et al 2007; Lartillot et al 2016) may adequately accommodate this rate variation.

The rate of substitution can also be calibrated by an outside source, such as a fossil or a known biogeographic break. These calibrations are often in the form of node constraints (Bromham et al. 2017). However, the placement of the constraint can have an impact on the estimated age and rate of evolution (Sauquet et al 2012; Duchêne et al., 2014). Duchêne et al. (2014) recommended including as many fossil calibrations as possible, with the deeper ones being preferred. However, Brown and Smith (2018) showed that more doesn’t necessarily equal better, as the interactions between the tree prior and node priors can cause deviations from the user-specified ages.

To illustrate the impact of spatial structure on tree variance and branch-length estimation, we randomly selected one genome from each species of the 6-taxon species tree vicariance simulations. These models have a uniform $T_{D}$ of 10,000 generations except for the most recent divergence between A and B, which occurred 5,000 generations in the past. After applying neutral mutations in *msprime*, we output all SNPs in FASTA format. To estimate the species tree, we used two inference programs, *BEAST2 (Ogilvie et al 2017) and SNAPP (Bryant et al 2012). For *BEAST2, we first created pseudo-genes by concatenating SNPs in intervals of 500 for the first 5000 in the genome for a total of 10 pseudo-genes (see Supplementary Materials for additional discussion about the use of “SNPs” with phylogenetic programs). These are “pseudo” in the sense that while their topology and branch-lengths tend to be correlated, they are not completely linked – what Springer and Gatesy (2016) call *concatenalescence*. While not ideal, this is standard practice for almost all phylogenomic datasets as the actual length of a “*c*-gene” may be as small as 10 bases (Springer and Gatesy 2016). The dataset for SNAPP consisted of 10,000 randomly selected SNPs from across the genome. Unlike *BEAST2, SNAPP does not estimate gene trees directly, instead iterates over all possible gene trees from biallelic SNPs to generate a species tree (Bryant et al 2012). In *BEAST2, we performed two separate runs per dataset: 1) with the root-age calibrated; and 2) with the clade ((AB)C) calibrated – or the “midpoint” calibration. We were particularly interested in differences that arose between the clustered (σ=0.25) and unclustered (σ=1.0) models relative to estimated clock rates, tree heights, and topologies. We visualized the estimated clock rates and calibration priors using Tracer v1.4.3 (Rambaut et al. 2018), and incongruence between estimated ages and topologies using the package DensiTree (Bouckaert 2010). In addition, we performed non-metric multidimensional scaling (NMDS) in R on sets of 1000 randomly selected trees from the posterior distribution. These trees were compared using weighted Robinson-Foulds (RF) distances, which account for both topology and branch length (Robinson and Foulds 1979). Finally, we compare the variance in estimated trees with the true gene tree variance by randomly sampling 1000 true gene trees, generating a weighted RF distance matrix, and plotting them using NMDS.

**Fig. 3.**
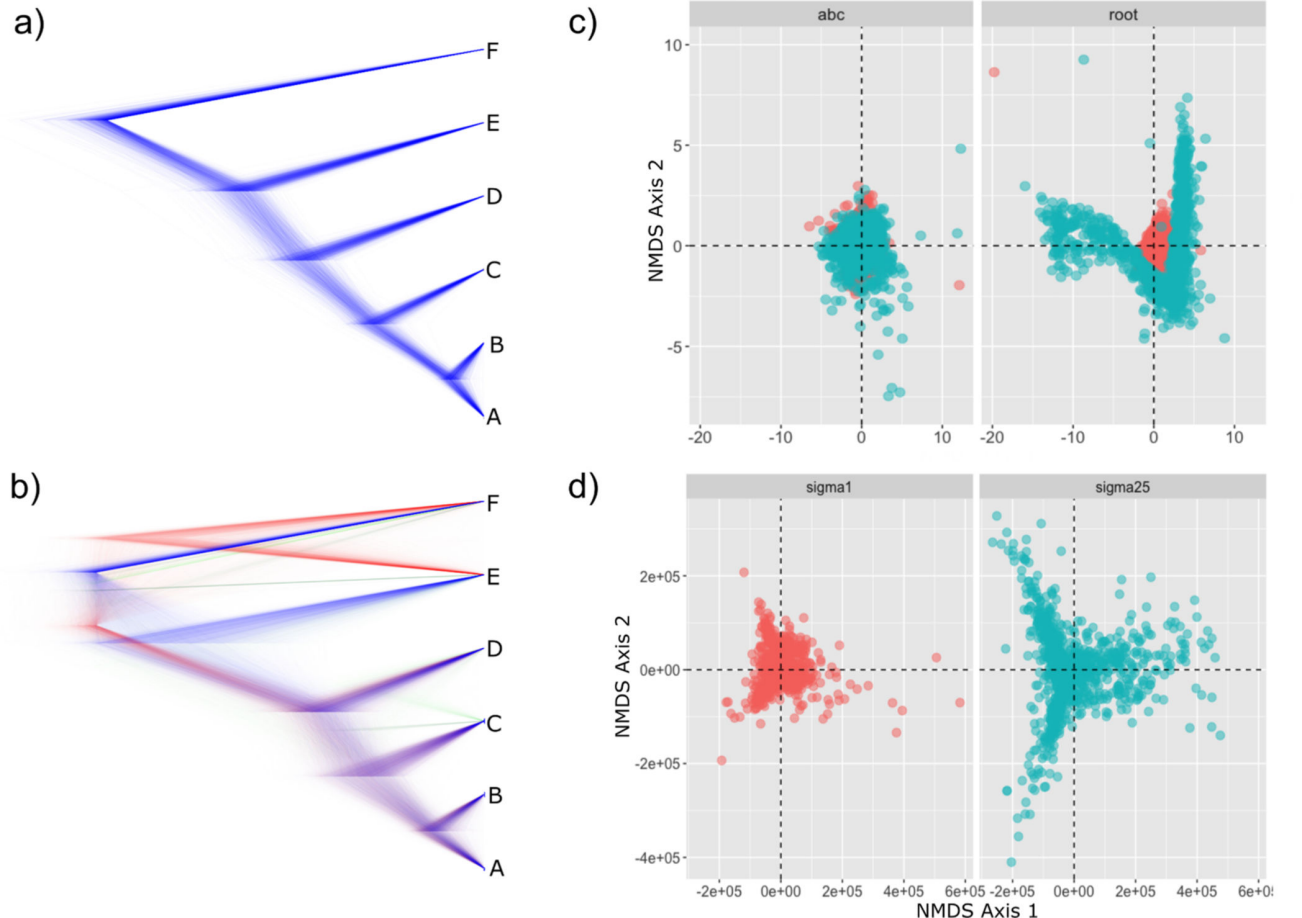
Gene tree variance. A) reconstructed gene trees from the unclustered model (sigma1); B) trees from the clustered model (sigma25); C) variance in topologies and branch lengths based on weighted RF distances, where “abc” is midpoint calibration and “root” is root calibration; D) true gene tree variance based on weighted RF distances.

**Fig. 4.**
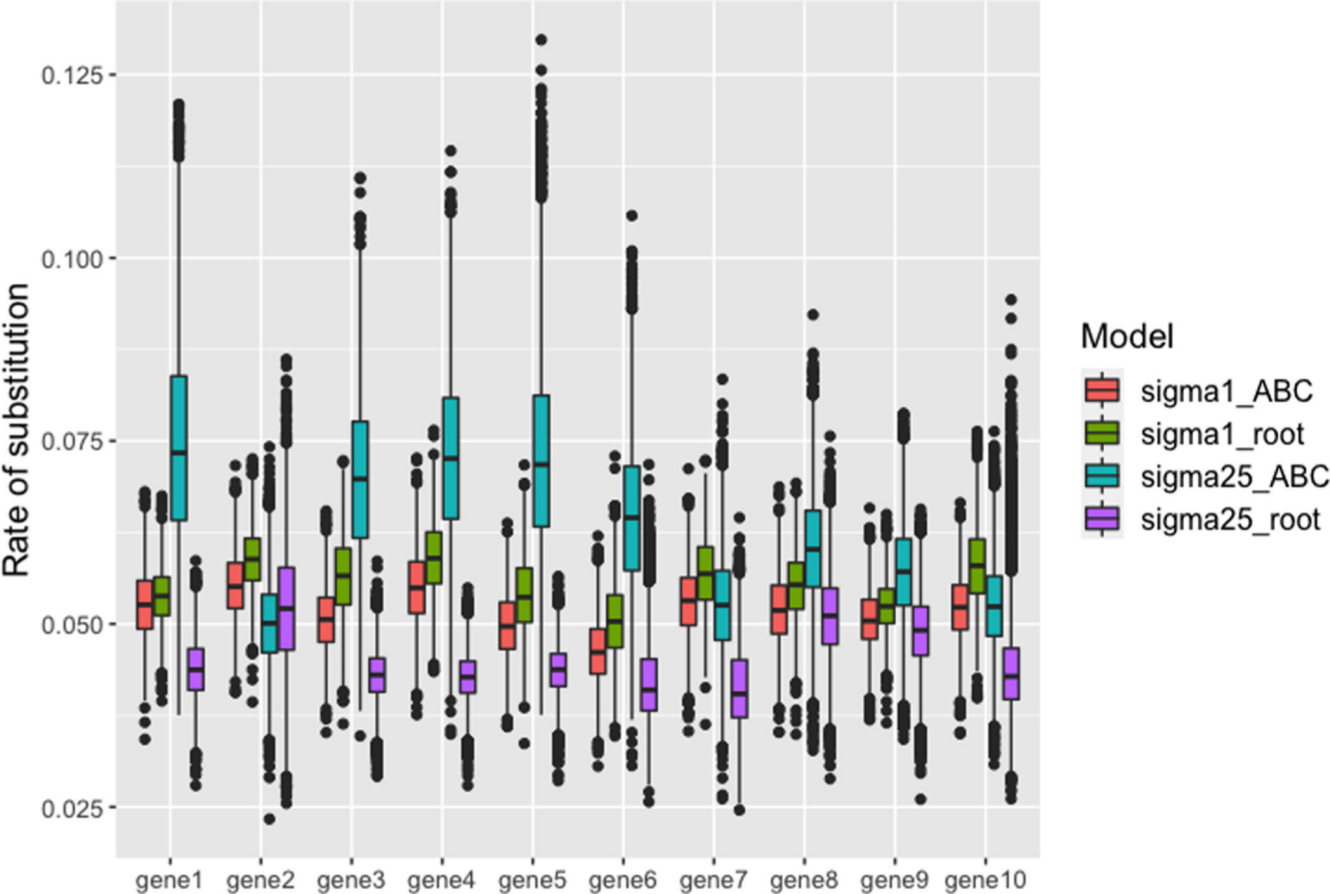
Substitution rate estimates from *BEAST2 for 10 pseudo-loci. For each model, “sigma1” is the unclustered and “sigma25” is the clustered, with “ABC” and “root” being whether the model was calibrated at the midpoint or the root, respectively. In general, for the unclustered models we find little variation between rates estimated from root or midpoint calibrations; however, when strong spatial structure is present (as is the case for the clustered model, sigma25), we find dramatic differences in rates depending on how the tree is calibrated with midpoint calibration resulting in much higher estimated rates.

The results from these analyses demonstrate the stark differences that emerge when spatial structure is strong (Fig. 3). We found that spatial structure greatly increased the variance in both the true gene trees (Fig. 3d) and the trees sampled from the posterior distribution when calibrated at the root (Fig. 3c). However, for the *BEAST2 analyses, constraining the lineages (ABC) to be monophyletic reduced the inferred species tree variation as shown in Fig. 3c; the clustered and unclustered distributions largely overlap each other in tree-space. In addition, the species tree inferred from the midpoint calibration was incorrect with topology ((((AB)C)D)(EF)). While the rooted calibration model generated the correct topology, node support was much lower than the unclustered models, which all had posterior probabilities of 1.0 (Fig. S4). The placement of the calibration impacted the estimated clock rate in both the clustered and unclustered models, but the rate differences between root and midpoint placements for the clustered model were far more extreme (Fig. 4). Irrespective of calibration placement, the 95% highest posterior density (HPD) of the node ages all included the true age for the unclustered models. For the root-calibrated clustered model, the true ages were obtained for all nodes, but the variance in the age estimate was much higher than in the unclustered model (Fig. 4). However, when the calibration was placed at the (ABC) node, the clustered model failed to identify the true ages for several nodes, and actually underestimated the root age by 22–60%.

The SNAPP analysis, despite being designed to handle biallelic SNPs, performed much poorer. Both the clustered and unclustered analyses produced aberrant topologies with high posterior probability, indicating that any violation of the panmixia assumption will bias species tree inference in SNAPP. However, estimates of θ generally followed expectations, inferring higher values for the ancestral branches of the clustered versus the unclustered (Fig. S5). In addition, the estimated tree height was greater in the clustered compared to the unclustered model (Fig. S5).

Degnan and Rosenberg (2006) and Degnan and Rhodes (2015) coined the term “wicked forests” to refer to the curious case where a set of species trees are one another’s most likely anomalous tree. In this section, we use the term more colloquially to refer to the expanded tree-space (or “forest”) that grows as spatial structure increases. These forests are “wicked” because they reduce our confidence in substitution rate, divergence-time, and topology estimates in species tree inference. Importantly, as was shown previously by Duchêne et al. (2014), the placement of the node calibration can rival the impact of spatial structure, and when placed at the midpoint of the tree actually masks the true gene tree variance. This masking also appears to reduce the ability of *BEAST2 to accurately infer the species tree. Since node calibration relies on constraining monophyly, the MCMC is limited in the space of trees it searches and therefore does not have a holistic sampling of the extent of ILS.

## 5. Species delimitation delimits structure

Identifying species boundaries is at the heart of systematics. The collection of techniques that fall under the category of “species delimitation” all aim, fundamentally, to distinguish between population structure and true species boundaries. These methods include four broad categories: 1) traditional morphological taxonomy; 2) distance-based methods; 3) tree-based methods; and 4) coalescent methods. It has been recommended to adopt a kind of holistic, consensus approach by considering results from each in deciding how robust the delimitation is (Carstens et al. 2013). The rise of the latter three methods coincides with increased sequencing effort for non-model organisms and the coincident “taxonomic impediment” (Lipscomb et al. 2003; Scotland et al. 2003). Each of these methods rely on a phylogenetic species concept (de Queiroz 2007) since they do not explicitly examine reproductive barriers – instead, these are inferred from an absence of gene flow between groups of samples.

Sukumaran and Knowles (2017), by simulating speciation under the protracted speciation model (PSM), were the first to point out that coalescent methods conflate lineage splitting with speciation. These results were replicated with an empirical system by Chambers and Hillis (2020), who showed that coalescent methods over-split species in the case of geographically widespread taxa. Leaché et al. (2019) respond by noting that the use of the PSM by Sukumaran and Knowles (2017) assumes instantaneous speciation, and that due to this feature no method would be capable of distinguishing population splits from speciation. Other studies have examined the impact of population structure directly on coalescent delimitation performance using stepping-stone simulations (Barely et al. 2018; Mason et al. 2020). These studies found, in accordance with results from Chambers and Hillis (2020), that coalescent delimitation over-split in the presence of population structure.

Both coalescent and tree-based methods were recently reviewed by Luo et al. (2018) under a series of speciation scenarios. They found that BPP (Rannala and Yang 2003) – a common coalescent software – generally performed better than the two tested tree-based methods, the General mixed Yule coalescent (GMYC; Pons et al. 2006) and Poisson tree processes (PTP; Zhang et al. 2013). However, their models of speciation did not incorporate population structure, only gene flow following speciation. Talavera et al. (2013) investigated the performance of the GMYC on a large-scale butterfly dataset and found that it often overestimated the number of species relative to the recognized morphospecies. Papadopoulou et al. (2008) found that the GMYC was only impacted by population structure under an island model of migration when the product of the population size and migration rate, Nm, was *<* 10^−5^. However, Lohse (2009) showed that by decreasing the number of demes sampled the GMYC would over-split even at higher migration rates.

In the previous section, we showed that low dispersal increased the variance in the $T_{MRCA}$ between species. In the context of species delimitation, where you may not know whether you’ve sampled from different species or merely different sites across the range of a single species, the variance in $T_{MRCA}$ within a single range becomes important. This variance is poorly captured by discrete population models but is an emergent property of continuous landscapes with finite edges. Samples taken from the center of the range on average share an ancestor deeper in the past than those from the periphery (Wilkins 2002; Wilkins and Wakeley 2004). This is the result of an increased population density in the center relative to the range edges. How does this variance in $T_{MRCA}$ across sampling locations impact species delimitation? Species delimitation methods, especially tree-based methods, rely on recognizing a shift in the coalescent dynamics of samples that are within versus between species. Spatial structure can increase the probability of monophyly between sampling locations, which makes them resemble discrete population clusters. The height of the node separating these clusters will dictate whether species delimitation methods identify them as separate species or merely clusters of samples within a single species. When samples are from the periphery, the distance between the clusters will be greater because each cluster, on average, shares a most recent common ancestor more recently than samples taken from the center of the range (Fig. S1–2). We contend that this feature of the coalescent in continuous populations demonstrates the importance of considering not merely the distance between two samples or the number of sampled sites, but whether those samples come from the core or the periphery of the range.

While these shortcomings have been identified for coalescent and tree-based methods, we contend that *all* species delimitation methods – including morphology-based – can be fooled by spatial structure. Unlike Sukumaran and Knowles (2017) and Leaché et al. (2019), we focus here solely on the ability of tree-based and coalescent-based models to distinguish population splits (i.e., we make no assumption that speciation has occurred).

**Fig. 5.**
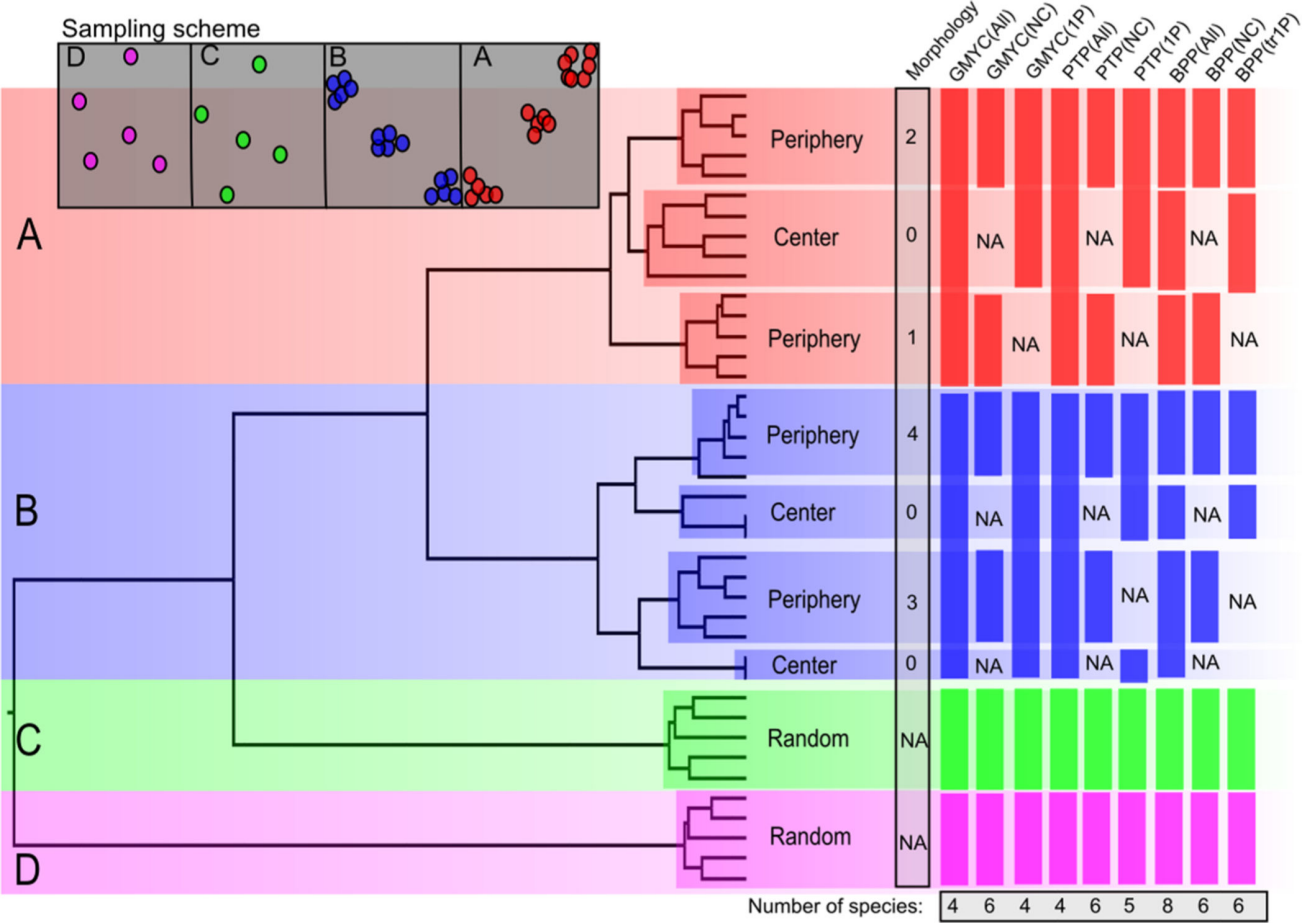
Species delimitation in continuous populations. Top-left boxes represent the sampling scheme, and the tree showed includes all samples, with clades colored by the species they belong to. Bars on the right are the inferred number of species from each method. “All” designates all samples included; “NC” is no center; “1P” includes a single periphery sample with the center. For the Morphology section, numbers beside clades are the number of simulated binary traits on the complete tree that support that clade as distinct from the rest of its conspecifics.

To investigate the performance of species delimitation methods on sequence data that arises from continuously distributed populations, we first sampled across the range of our simulated 4-taxon species tree vicariance models for both the clustered and unclustered settings. For species A and B, we sampled 5 individuals each from three locations, including two edges and the center (Fig. 5). For species C and D, we randomly sampled 5 individuals across the range. We then randomly sampled 10,000 SNPs from the genome alignment and constructed an ultrametric tree using BEAST2 with a birth–death tree prior. We generated consensus trees using TreeAnnotator and imported these trees into the R platform. For the GMYC method, we used the package *splits* (Fujisawa and Barraclough 2013) to calculate the ML estimate for the transition between a Yule and coalescent model. For the PTP method, we used the web server (https://species.h-its.org/ptp/) and both a ML and Bayesian estimation of the number of splits in the tree. For the coalescent-method, we used BPP, which can jointly infer the species tree and perform species delimitation using a reversible-jump MCMC algorithm (Rannala and Yang 2013). For BPP runs, we generated 5 pseudoloci (see section Wicked Forests: Species Tree Inference and Gene Tree Variance) of 500 SNPs. The MCMC was run for 100,000 generations with a 20,000 generation burn-in. To illustrate how morphological delimitation can be deceived in the presence of population structure the same as tree- and coalescent-based methods, we simulated 20 binary traits onto the phylogeny produced by BEAST2 with *phytools* (Revell 2012) in R under a Brownian-motion model. The *Q*-matrix was defined such that there was an equal transition probability between trait 0 and 1. We then counted traits that clustered by sampling location as opposed to by species.

Delimitation studies aimed at identifying species, especially in the case of cryptic species complexes, generally rely on a consensus approach to interpret their delimitations. In Fig. 5, we see that *all* delimitation methods can be misled by spatial structure when sampling is biased towards low diverse areas of the range (such as the periphery). Furthermore, if we were to search for specific morphological traits to support our delimitations, we have a good chance of finding at least 1 trait (Fig. 5). The specific sampling scheme also has a clear impact, but not necessarily because some samples were random whereas others were clustered. Instead, what matters is where the samples are collected in respect to the local population density. When samples are drawn from the periphery, they will have shorter coalescent times and therefore longer branches to other samples from the same population. However, drawing samples from the center of the range, where the local $N_{e}$ is the highest, largely ensures that deeper node heights will be included. These deeper heights reduce the clustering pattern and the probability of monophyly. For example, the B species samples from the center break the monophyly between the peripheral populations (Fig. 5). In the case where all three samples are monophyletic, as in species A, the deep node heights of the center samples are sufficient to allow the tree-based methods to accurately identify the transition between Yule and coalescent models. Removing these central samples accentuates the clustering pattern, and consistently fools both tree-based and coalescent-based methods. The geographic locations were not monophyletic in the unclustered model and the node heights on the inferred tree were as deep as the random samples. Therefore, we did not perform the tree-based methods as no empirical study would use these methods in the absence of any geographic clustering.

While the tree-based methods performed better when all samples were included, BPP consistently overestimated the number of species. The species number with the highest posterior probability in each run matched the number of geographic samples, regardless if the center samples were included or absent (Fig. 5). In the unclustered model, BPP identified 5 species (PP = 0.598) when the guide species tree included all 8 geographic locations. This was much closer to the true 4 species total than in the clustered model.

Unlike previous examinations of species delimitation methods in which independent lineages may be designated as “lineages” or as *bona fide* “species” (Sukumaran and Knowles 2017; Leaché et al. 2019), we only assess the ability of these methods to identify actual population splits irrespective of if reproductive isolation had occurred. In addition, by using a continuous landscape we are better able to assess sampling schemes and how variation in local population density impacts delimitation methods. In contrast to Luo et al. (2018), we find that the tree-based methods outperform BPP when samples from the center of the range are included, and that of those the GMYC consistently converged on the true number of species (Fig. 5).

## 6. Out of space: continuity for discontinuous data

In a review of Battey et al. (2020) for the *Molecular Ecologist*, Jeremy Yoder described continuous space as the “final frontier” for population genetics (Yoder 2020). In this article, we extend Yoder’s proclamation to phylogenetics as well. As continuously distributed populations impact virtually all commonly used summary statistics in population genetics (Battey et al. 2020), we show above that it also affects broader macroevolutionary patterns. Each of these patterns have been discussed in the literature previously (e.g., Edwards and Beerli 2000; Slatkin and Pollack 2009; Sukumaran and Knowles 2017; Hancock and Blackmon 2020) but have not explicitly been tied to continuous populations or with a focus on the mode of speciation. Below, we review the preceding sections by answering three questions that empiricists should consider when conducting phylogenetic inference.

### 6.1. When does space matter for phylogenetics?

Answer: When spatial autocorrelation of ancestry persists between speciation intervals. This is most likely to occur when there is heterogeneity in population density across the range, low dispersal or local retention, and speciation intervals are short relative to ancestral $N_{e}$.

As discussed in *Slatkin’s Skew and Gene Tree Asymmetry* above, spatial autocorrelation of ancestry decays with time at a rate dictated by the size of the range and the rate of dispersal. Given enough time, the ancestors of a sample of individuals will appear as a random sample across the range and the rate of coalescence will collapse to Kingman’s coalescent (Kingman 1982; Wakeley 2009). To provide a simple illustration of this, imagine a Markov process with transition matrix.

$$P^{t}={\left[\begin{array}{ccc} a_{a} & a_{a\to b} & a_{a\to c}\\ b_{b\to a} & b_{b} & b_{b\to c}\\ c_{c\to a} & c_{c\to b} & c_{c} \end{array}\right]}^{t}$$

where a, b, and c represent transition probabilities to either different states (above and below the diagonal) or remaining within the currently occupied state (along the diagonal). As a concrete example where t=1:

$$P^{1}={\left[\begin{array}{ccc} 0.99 & 0.01 & 0.0\\ 0.01 & 0.98 & 0.01\\ 0.0 & 0.01 & 0.99 \end{array}\right]}^{1}$$

In this example, there are three demes represented by each row in the transition matrix. The transition probabilities can be thought of as dispersal rates between demes. Clearly, when *t* is small the ancestors of any deme will be biased towards the deme that they were originally sampled from. For example, when t=5:

$$P^{5}={\left[\begin{array}{ccc} 0.99 & 0.01 & 0.0\\ 0.01 & 0.98 & 0.01\\ 0.0 & 0.01 & 0.99 \end{array}\right]}^{5}=\left[\begin{array}{ccc} 0.952 & 0.047 & 0.001\\ 0.047 & 0.906 & 0.047\\ 0.001 & 0.047 & 0.952 \end{array}\right]$$

While spatial autocorrelation is still strong at t=5, we see that there is now a non-zero probability of a lineage originating in deme 1 having an ancestor in deme 3, and vice-versa. This toy example also shows that central demes spread out to the periphery much faster than peripheral populations reach one another, which indicates that if the sample originates from the center of the population the transition to the scattering phase may be faster than for samples from the periphery. How much faster? In our example, by t=45 (or 45 generations before the present), samples from the center have a 50:50 chance that their ancestor was also in the center deme. Alternatively, the peripheral demes each have ~ 70% probability that their ancestors at generation 45 are still found in the sampled deme.

The transition to the collecting phase occurs as the probability that a sampled individual has an ancestor in any deme is equal. In fact, Wakeley (1999) defined the end of the scattering phase as the *t* when all ancestral lineages are in separate demes (i.e., they are scattered evenly across space). Continuing with our example, this would occur when the transition probabilities are all ~ 33%, which does not occur until t≈400. In a phylogenetic context we might imagine one of the peripheral demes has a complete barrier to gene flow with the others until ${\text{T}}_{\text{D}}$. At this time, bias in tree topologies and branch lengths will occur if ancestors are still correlated with their sampled deme. In this case, if the true tree is ((Center, Periphery1), Periphery2), we would expect a skew in topologies towards ((Center, Periphery2), Periphery1) over ((Periphery1, Periphery2), Center), where the MSC would predict they should occur with equal probability. Furthermore, trees that do show the latter topology will be biased towards older node heights since it on average takes longer for the edges to find ancestors with one another.

Clearly, the rate of transition between the scattering and collecting phases is a function of the number of demes and the rate of migration between them. While this is much more complex in continuous space (there are more dimensions to transition through, for example), this simple example can serve to prime our expectation of when space will matter in macroevolutionary studies. Also, important to note is that the above example is independent of $N_{e}$, which is the parameter that actually determines the rate and average times to coalescence in real populations.

### 6.2. What sort of organisms will be the most affected?

Answer: Those with large ranges relative to their dispersal potential with a history of vicariance. A famous example from the biogeography literature is the vicariance event that impacted coastal marine organisms separated by the Florida peninsula (Bert and Harrison 1988; Bowen and Avise 1990; Avise 1992; Flannagan et al. 2016). While several hypotheses have been purported to explain the cause of vicariance in these marine taxa (see Portnoy and Gold 2012; Hancock et al. 2019 for recent reviews), sister species are otherwise widespread with varying degrees of population structure. Species that show low structure within populations, such as oysters (*Crassostrea virginica*) with pelagic dispersal, may approximate our “unclustered” models above, which show little to no bias on phylogenetic inference. Alternatively, species such as brooding amphipods (Hancock et al. 2019), which have poor dispersal potential and strong population structure, are more akin to our “clustered” models and are expected to have high gene tree discordance, increased variance in node heights across the posterior sample, and likely upwardly biased estimates of species diversity (all of which were documented in Hancock et al. 2019). Another example is the Gulf pipefish (*Sygnathus scovelli*), which displays population structure within species as well as discrete barriers between those isolated by the Mississippi River and the Florida peninsula (Flannagan et al. 2016).

### 6.3. How can we detect if spatial structure is influencing our phylogenies?

Answer: Fortunately, our data contain a myriad of clues into a history of spatial structure. At the phylogeographic level, in which researchers are often examining species complexes or closely related taxa with broad geographic sampling, signals of vicariance and continuous structure can be quite obvious. Hancock and Blackmon (2020) evaluated the impact of isolation-by-distance on phylogenetic inference in a species complex of Brazilian endemic squamates. Despite the ability of species delimitation methods to identify discrete clusters (Domingos et al. 2017), when samples were drawn from the range edges the estimated ages were upwardly biased compared to neighboring localities (Hancock and Blackmon 2020). This pattern can only emerge if the range was once widespread with ancestral IBD.

The impacts of space will also lead to high variance in reconstructed topologies and low posterior probabilities, especially at deeper nodes (Fig. 4). A model of ILS alone is sufficient to predict the topological incongruence in Fig. 4 – i.e., we expect some of the most common trees that are incongruent with the species tree to be ((((AB)C)D)(EF)) and ((((AB)(CD))E)F) – but it does not explain the high variance in reconstructed node heights. Furthermore, when the calibration is placed at the root, we find that the increased variance in both topologies and node heights in the posterior emerge as a result of the same variance in the underlying true gene trees. While there are many factors in practice that can increase variance, such as low information content, sequencing error, etc., the most fundamental is the true gene tree variance. Assuming that other sources of variance can be isolated and accounted for, recognizing this underlying gene tree structure could be an indirect way to peak at the demographic processes responsible for patterns in the data. A possible test of spatial structure would then be to examine the tree-space of reconstructed topologies and branch-lengths from the posterior. A peculiar pattern that emerges when examining the spread of trees in multidimensional space is that strong spatial structure pulls the mean tree away from the origin in both the true trees (Fig. 4d) and the reconstructed trees (Fig. 4c).

Another promising avenue may be to compare the impact of node calibration placement on gene tree variance and rate estimation. While both the unclustered and clustered models showed some impact of calibration placement, the clustered model resulted in extreme differences between rates estimated with different placements (Fig. 5). In addition, like the topologies and branch-lengths mentioned above, the variance in these estimates were much higher than in the unclustered models. Thus, a side-effect of strong spatial structure is reduced confidence in rate estimation, topological inference, and branch-length estimation, even when incorporating node calibrations that include the true age. We recommend researches not jointly infer substitution rates and topology; instead, first reconstruct the species tree and then infer rates on the fixed tree.

Gene tree stoichiometries are important sources of information about the underlying demographic histories of populations but can be misleading if not considered within an explicitly spatial context. In the case of a 4-taxon tree, one could investigate the possible impact of space by first summing the proportion of trees that include D (or the outgroup) as the ingroup (as shown in Fig. 2c). Next, one could regress the physical distance between D and its sister species for a given tree against the total proportion of gene trees. Significant negative relationships between topological proportions and distance indicate that gene tree topologies may be driven by historic spatial relationships. This does not preclude hybridization, but it does demonstrate that a more robust criteria must be adopted to distinguish spatial structure from introgression.

**Fig. 6.**
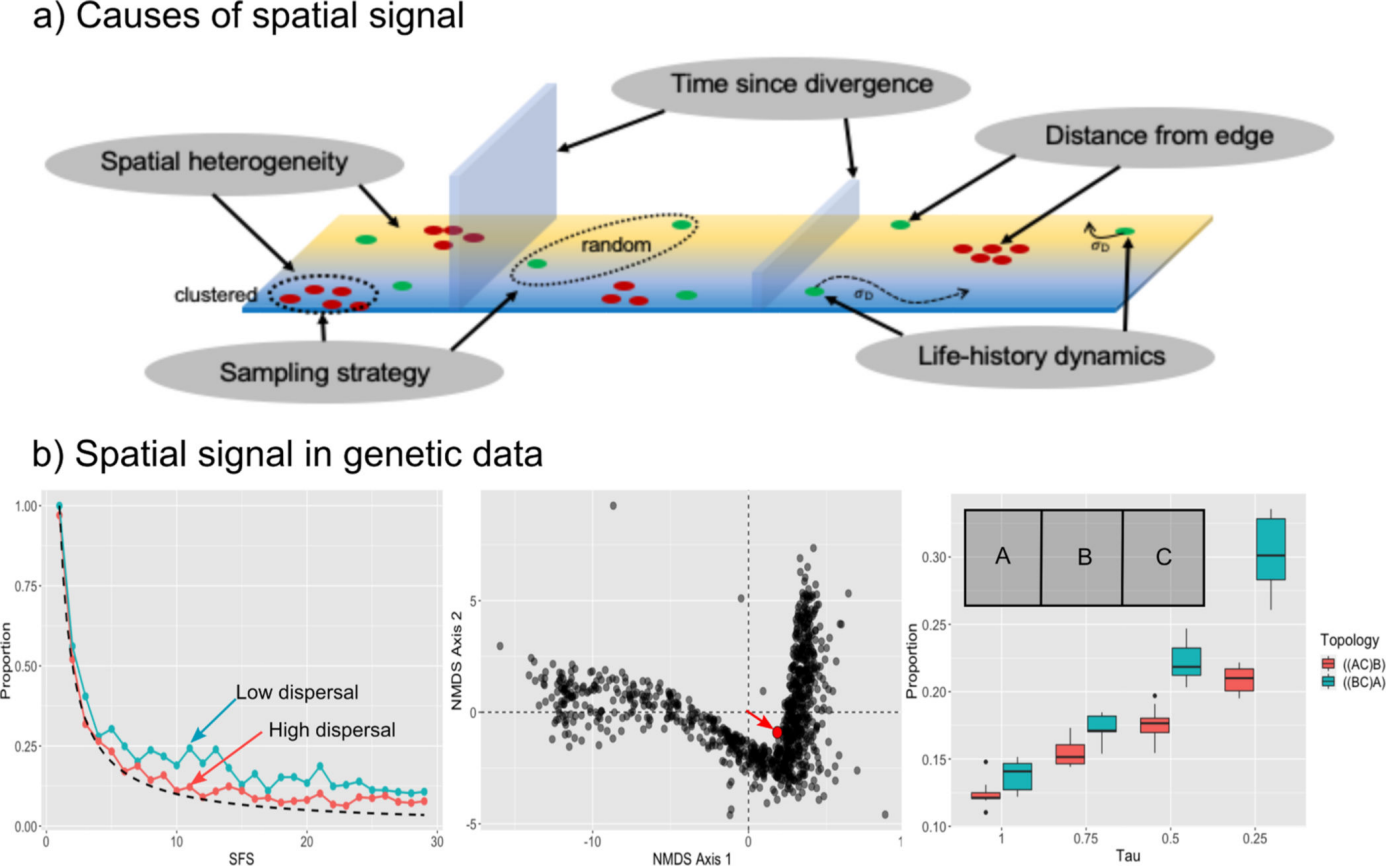
How space impacts phylogenetic datasets. A) a schematic of the different causes of spatial signal; B) specific signals that can be found in phylogenetic data. From left to right: skews in the SFS at lower dispersal; pull of the mean tree away from the origin in NMDS space; skews in gene tree topologies that reflect a vicariant history.

Theunert and Slatkin (2017) provided a method for distinguishing between ancestral population structure and admixture using the unfolded site frequency spectrum (SFS). They showed that structure causes a skew from the theoretical expectation of $\frac{\text{θ}}{i}$ towards intermediate frequency alleles, whereas admixture did not alter the SFS. We recapitulate their results in Fig. 6b and show that the unclustered model leads to much less skew in the SFS than the clustered. In a phylogenetic context, the SFS could be used in conjunction with methods that reconstruct ancestral areas, such as RASP (Yu et al. 2015), to distinguish ancient hybridization from continuous spatial structure. This could be done via model comparison in a Bayesian framework by conditioning a model of hybridization versus spatial structure on the shape of the SFS and the probability of hybridization given reconstructed ancestral areas.

In many respects, ancestral ranges actually confound our ability to distinguish structure from hybridization as individuals need to be in geographic proximity to hybridize in the first place, but it is this very proximity that makes structure a likely explanation for geographically skewed topologies. Ideally, we would weight the posterior probability of a model of hybridization versus structure by incorporating the SFS as a prior. But what about when hybridization *and* structure characterize the history of our samples? In this case, we expect a skew in the SFS towards intermediate frequency alleles in conjunction with overlapping ancestral areas, exactly as predicted by continuous structure alone. However, we could leverage simulations over various histories of admixture proportions in conjunction with spatial structure to determine which combinations of models are identifiable. For example, for periods of strong admixture in the distant past we might expect to see skews in gene tree topologies that exceed that predicted by spatial structure alone. Indeed, if hybridization was prevalent enough and occurred $>2N_{e}$ generations in the past, the influence of spatial structure may be largely washed-out and most, if not all, of the skew should be caused by hybridization (Fig. 2).

The subfield that may be most improved by considering spatial structure is species delimitation. There exist several methods in spatial population genetics whose underlying structure could be co-opted to improve existing techniques. For example, Bradburd et al. (2018) introduced an R package, *conStruct*, which seeks to identify both discrete and continuous population boundaries by modelling an expected decay in allelic covariance with distance. Like STRUCTURE (Pritchard et al. 2000), *conStruct* accepts a user specified number of layers, K, but unlike STRUCTURE allelic covariance is allowed to decay with distance within a single layer. To incorporate geographic distance and the covariance within layer k of allele i and j, they use.

$$G_{ij}^{\left(k\right)}=\alpha_{0}^{\left(k\right)}\left(\exp\left(-{\left(\alpha_{D}^{\left(k\right)}D_{ij}\right)}^{\alpha_{2}^{\left(k\right)}}\right)\right)+\phi^{\left(k\right)}$$

where $\alpha^{\left(k\right)}$ parameters dictate the decay of covariance and $D_{ij}$ is the geographic distance between sample i and j, and ${\text{ϕ}}^{\left(k\right)}$ is the background covariance within the entire layer (Bradburd et al. 2018). By setting $\alpha_{0}^{\left(k\right)}=0$ we recover the nonspatial model (i.e., k is governed by a single shared frequency parameter, ${}^{\left(k\right)}$). Bradburd et al. (2018) use a cross-validation approach to compare the spatial and nonspatial models. In this way, one can imagine the K with the highest support representing the maximum number of species present in a sample while accounting for the expected decay of relatedness with distance. This model could be extended to incorporate time separating the K discrete layers as allelic covariance is expected to decay across both space and time. Thus, for more distantly related species this decay will be less influenced by space than by time, and higher numbers of K should be preferred.

### 6.4. A future in space

The field of phylogenetics has taken the form of an hourglass. At one bulge is phylogeography, in which multiple geographic locations and individuals within a species are sampled. Phylogeographers straddle the boundaries between population genetics and phylogenetics, plucking methods from both to query the recent past of relatively closely related individuals. At the opposite bulge is deep-time phylogenetics that is investigating the origins of families, orders, or even phyla, where the ancestors of sampled individuals lived on a landscape dramatically different from the one today. These phylogenies may have hundreds of tips, but each species is often represented by a single individual, and tips are not related to one another in space. Both ends of the hourglass have seen tremendous developments in the past decades thanks to improved sequencing technology and more computationally tractable methods for dealing with large numbers of tips. However, both tend to use phylogenies as only informative about time.

We contend that the next important steps in phylogenetics will not merely be in time, but in space. By incorporating the z-axis into our models, we can better identify the mode of speciation and characterize the demographic factors influencing our reconstructed trees. Furthermore, by adopting a spatial framework conceptually provides us with an intuition about gene tree variance and can caution our interpretations of widespread inferences of hybridization. In addition, considering space encourages us to visualize tree-space, which can lead to more rigorous scrutiny of consensus methods that often fail in the presence of population structure (DeGiorgio and Rosenberg 2016). Finally, we believe that spatial models of IBD can dramatically improve species delimitation methods by conditioning the inferred number of species on an expected decay of allelic covariance with distance.

## Supplementary Material

supplementary material
