# Supplementary material for: Phylogenetics in space: How continuous spatial structure impacts tree inference
Source: Mol Phylogenet Evol. Author manuscript; Available in PMC 2023 Jun 23. (PMC10289471; doi:10.1016/j.ympev.2022.107505)

***Supplementary Materials***

*Model details and discussion*

The continuous-space model is an extension of Rogan et al. (*in prep*), which itself was a modification of Bradburd and Ralph (2019) and Battey et al. (2020). Unlike in these previous applications, the model is constrained to non-overlapping generations by enforcing a pseudo-age structure, which sets fitness to 0 at age 1. Beyond this, the model is unlike the classical WF model in that population size is not constant but is an emergent property of the population carrying-capacity, *K*. Furthermore, while the mean and variance of the number of offspring should converge to a Poisson process with 𝝀 = 1 as *N* → ∞ (Wakeley 2009), in our model the population is only sustained when 𝝀 = 2. Therefore, the mean and variance in offspring is slightly higher than under the WF model.

The landscape is defined by a matrix, *M*, of height *h* and length *ls*, where

$\left\{ \begin{matrix} length\left( M \right)=ls, & \mathrm{when}s=1 \\ length\left( M \right)=ls+4s, & \mathrm{when}s>1 \end{matrix} \right\}$.

The inclusion of 4*s* provides a distance of 4 cells between each species, *s*, following the split while ensuring that the species-specific *M* = *h* ✕ *l*. This distance exceeds the maximum dispersal distance in the unclustered model. The number of species simulated were 3, 4, and 6, each with different applications. We used interpolation to allow the fitness values of cells to be a continuous gradient instead of discrete steps between cells. The cell-specific fitnesses, *f_c_*, were equal to 1.0 (i.e., there were no cell-specific fitness declines).

Fitness was also determined by the local population density with a maximum strength of 1 / 2π𝜎_I_^2^ and max distance of 3𝜎_I_, which was drawn from a Gaussian distribution. To reduce clumping along the edges, we enforce fitness decline relative to distance from the edge as

$f_{e}=\min\left( 1, \sqrt{\frac{x_{i}}{\sigma_{D}}} \right)\min\left( 1, \sqrt{\frac{y_{i}}{\sigma_{D}}} \right)\min\left( 1, \sqrt{\frac{(M-x_{i})}{\sigma_{D}}} \right)\min\left( 1, \sqrt{\frac{(M-y_{i})}{\sigma_{D}}} \right)F$,

where *F* is the cumulative fitness accounting for both *f_c_* and 𝜎_I_, and *x_i_* and *y_i_* are the spatial positions of individual *i* in the *x* and *y* coordinates, respectively (Battey et al. 2020). This causes fitness declines towards the edges to be more extreme when 𝜎_D_ < 1.0, which promotes heterogeneity in population density across the range.

Individuals are hermaphroditic but incapable of selfing. Mate-choice strength is drawn from a Gaussian distribution with a maximum of 1 / 2π𝜎_M_^2^ within a max distance of 3𝜎_M_. Immediately after offspring production, individuals disperse across the range by taking two draws from a random uniform distribution with a minimum of –3𝜎_D_ and maximum of 3𝜎_D_. The two life-history models we refer to as “clustered” and “unclustered”, which characterize the behavior of the simulated individuals. The clustered model sets 𝜎_D_ and 𝜎_M_ each = 0.25, and 𝜎_I_ = 1.0; the unclustered model sets 𝜎_D_ and 𝜎_M_ = 1.0, and 𝜎_I_ = 0.25.

For the vicariant model of speciation, the initial range of size *M* is populated with *M*K* individuals, where *K* is the local carrying-capacity, with initial positions drawn from a random uniform distribution. For the 3 and 4 species models, a period of 10,000 generations of burn-in precedes the initial split. For the 6 species models, this period is extended to 50,000 generations. The range then proceeds through a series of fragmentations that carve the range into *s* equal final ranges of size 20✕20. For the peripatric speciation model, the fitness of the initial range is set to 0.0 except for the far-left corner of size 20✕20. The population then proceeds through an expansion period of 100 generations where the habitable range size has extended to a length of *ls* + 4*s*. After 100 generations, the range is split by setting the fitness values of 4 columns of the landscape matrix to 0.0. This occurs *s* times.

Simulated individuals are diploid with genome sizes of 1000 Mb and a recombination rate of 10^-9^ (on average, there will be 1 recombination event per individual per generation). No mutations are simulated during the runs as these can be overlaid after to increase computational efficiency. Tree-sequence recording is enabled to track the true local ancestry of all individuals; however, throughout the run internal simplification occurs that discards nodes that do not contribute offspring to the final generation.

Several important theoretical points about the model should be noted. First, both the clustered and unclustered models have a period of spatial autocorrelation and therefore neither represent true random mating. The unclustered model is merely meant to *approximate* random mating. Along these lines, the neighborhood sizes of each model are < 1000 and therefore will not generate random mating expectations (Wright 1943). Furthermore, in the clustered model the neighborhood size may be low enough that it *never* converges to the *n*-coalescent (Kingman 1982; Wilkins 2004; Wakeley 2009). Another way to state this is that there is no transition between the scattering and collecting phases – the population is locked in the scattering phase. However, the process of recapitation in *msprime* (see *Trees in Space: Model*) ensures that the collecting phase occurs as it simulates coalescence among all multiple roots under the *n-*coalescent irrespective of geography, life-history, etc. We justify this forced transition by pointing out that any uncoalesced nodes present at the beginning of the simulation either 1) belong to the same species, usually the last *k* = 2 samples to coalesce or 2) are from different species, and therefore represent a case of deep coalescence. The former case will add high frequency SNPs on the last long branches leading to the most recent common ancestor. These high frequency SNPs lead to deviations from the expected unfolded site frequency spectrum, *E*[𝜉*_i_*], which under neutrality should be $\frac{\theta}{i}$; we see an inflated proportion of high frequency SNPs in the site frequency spectrum (**Fig. 4.6**). Importantly, this occurs in both the clustered and unclustered models. However, as seen in **Fig. 4.6**, the clustered model leads to greater deviations in *E*[𝜉*_i_*] at intermediate frequencies. The impact of (2) on topological inference will be conservative as it will reduce the *T*_MRCA_ by forcing random mating.

*Identifiability of 4-taxon trees*

In the section *Slatkin’s Skew and Gene Tree Asymmetry*, we provide the results of both 3- and 4-taxon simulations. There’s an important point that should be noted regarding *t* in each of these models. Since *t* is in units of 2*N_e_*, to accurately reflect theoretical expectations of divergence times we need to know *N_e_*. For the 3-taxon tree, *t* can be calculated directly. However, for the 4-taxon tree there are an infinite number of combinations of *t*_1_ and *t*_2_ that produce a given proportion of gene trees. Therefore, the actual *t*_1_ and *t*_2_ values – those that reflect the “true” *N_e_* – cannot be identified. The lack of identifiability is twofold: 1) the infinite combinations issue already pointed out; and 2) that reducing *T*_D_ also reduces the amount of time a population has to equilibrate following the split. Therefore, estimating *N_e_* from π_12_ would be incorrect because the relationship π_12_ = 4*N_e_*𝜇 only holds if the population is in equilibrium. This is further exacerbated in the clustered model as it takes much longer to return to equilibrium following a split than the clustered model does. Due to these issues, for the 4-taxon tree we examine gene tree proportions in units of 2*N*, where *N* is the census population size. This means that the coalescent units in **Fig. 4.2c** between “sigma1” and “sigma25” are not the same. However, since we focus only on the topologies that are expected to be equal at all values of *T*_D_, we do not think this disparity impacts the results presented.

*SNPs and pseudo-loci*

Most widely-used phylogenetic programs (such as *BEAST2 and BPP, which are used here) rely on invariant sites for accurate substitution-rate estimation and generally assume that each locus in a multilocus dataset has a single gene tree topology. As discussed in the main text, the latter point is rarely the case (see Springer & Gatesy, 2016). Furthermore, while it is technically possible to concatenate SNPs and perform phylogenetic analyses as if they were a single locus, this can potentially lead to overestimating terminal branch-lengths (see <https://groups.google.com/g/beast-users/c/uDgbuT5-hnk/m/R2qykTe7BQAJ>). Despite this, we use what we call *pseudo-loci*, which are concatenated SNPs, as our input for *BEAST2 and BPP. We do this for a few reasons, explained below.

Firstly, the simulation software we utilized does not generate entire genomes for computational reasons (i.e., invariant sites are not retained). Instead, only SNPs, and their genomic locations, are retained and output. However, we can scan for topological congruence across SNPs because, in general, SNPs that are in physical proximity tend to fall on gene-trees that share the same topology even if technically recombination has occurred between them. In an empirical dataset where we did not know exactly where recombination had occurred, we could not distinguish these as separate *c*-genes.

Second, they are only SNPs in that they can vary between species but may not vary within species. This is due to the fact that SLiM does not simulate “species”, but populations and the underlying tree structure is a product of the splitting regime. Therefore, loci can be “fixed” within and between populations for different alleles and yet still be considered “SNPs”. Truly invariant sites, however, as noted above, are not retained.

Finally, and most importantly, none of the analyses performed here rely on an accurate substitution rate estimate (i.e., we make no assumption about what the *true* rate is); instead, we are merely interested in how differing strengths of spatial structure impact this estimate. For example, in **Fig. 5** we show that calibration point (midpoint versus root) results in dramatically different rate estimates between the clustered and unclustered models. If this was explained solely by the influence of biases due to concatenating SNPs, we would not expect this difference between models to emerge. Furthermore, despite the possibility of biased terminal branch-length estimates, the 95% HPD of all node ages in *BEAST2 for both models included the true age.

**Supplementary Figures**

**Figure S1**. Distribution of *N_e_* / *N* across the range for both clustered (𝜎 = 0.25) and unclustered (𝜎 = 1.0) models for the peripatric speciation scenario. Dotted line represents location of population split. Right panel is before the expansion; central panel is the expansion phase; left is after the expansion.


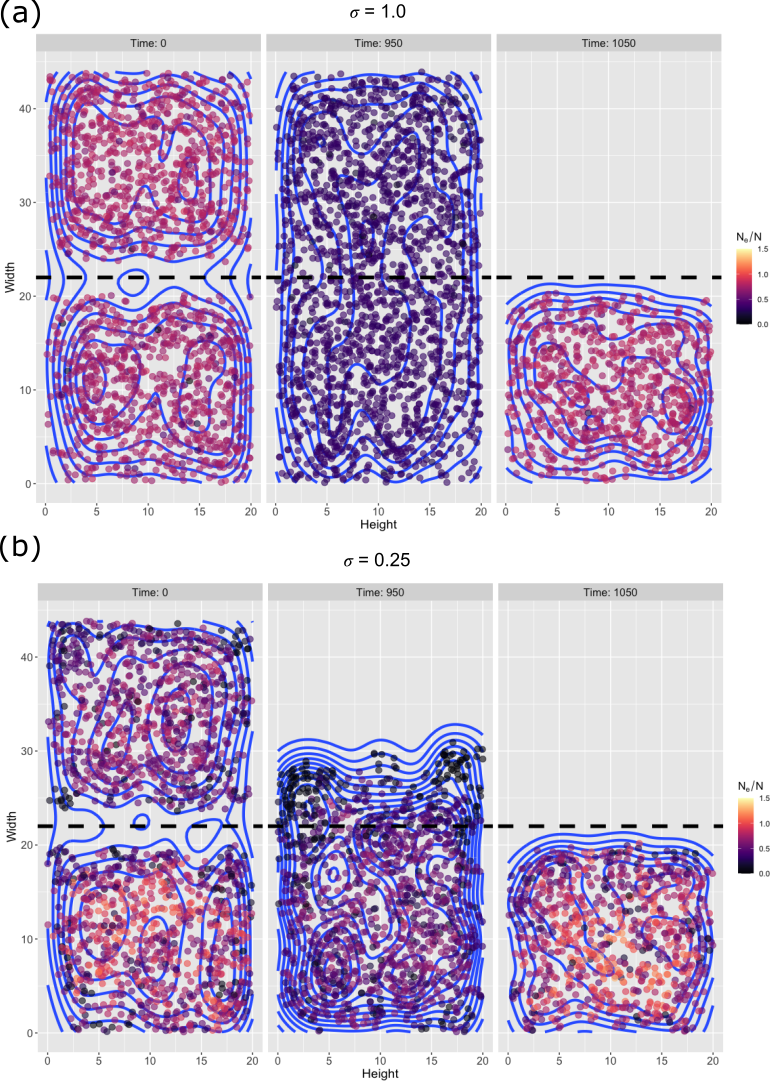


**Figure S2**. Distribution of *N_e_* / *N* across the range for both clustered (𝜎 = 0.25) and unclustered (𝜎 = 1.0) models for the vicariant speciation scenario. Dotted line represents location of population split. Right panel is prior to the split; middle is 10 generations after; left is 990 generations after.


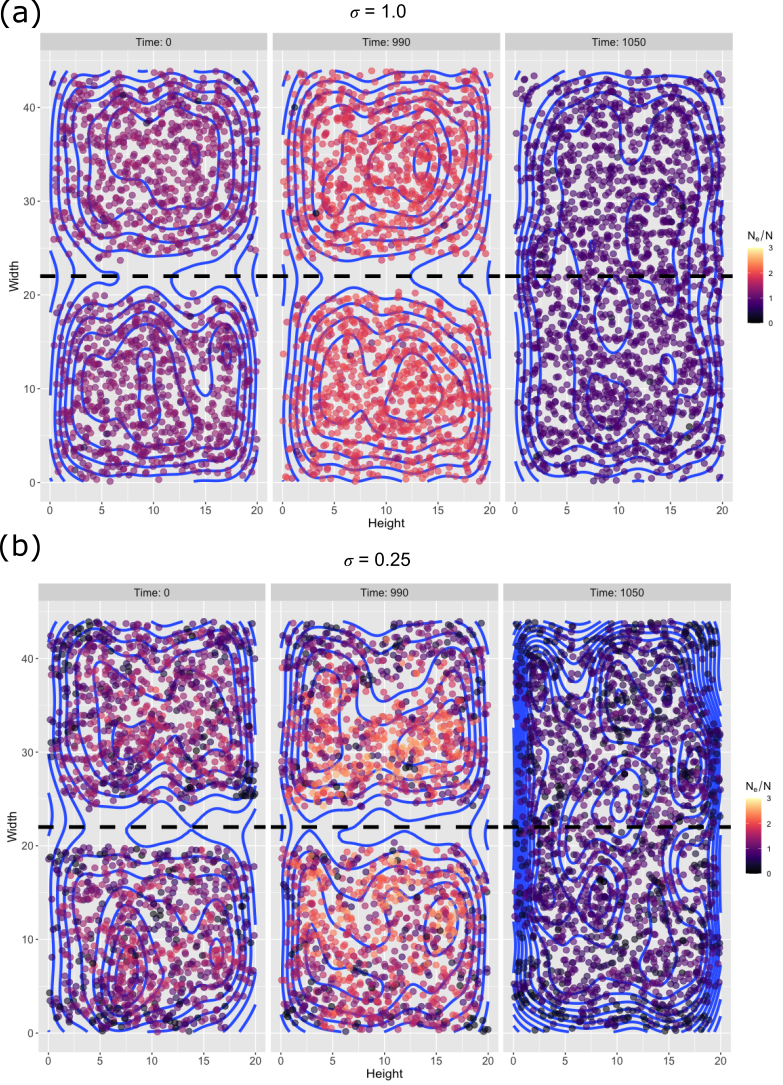


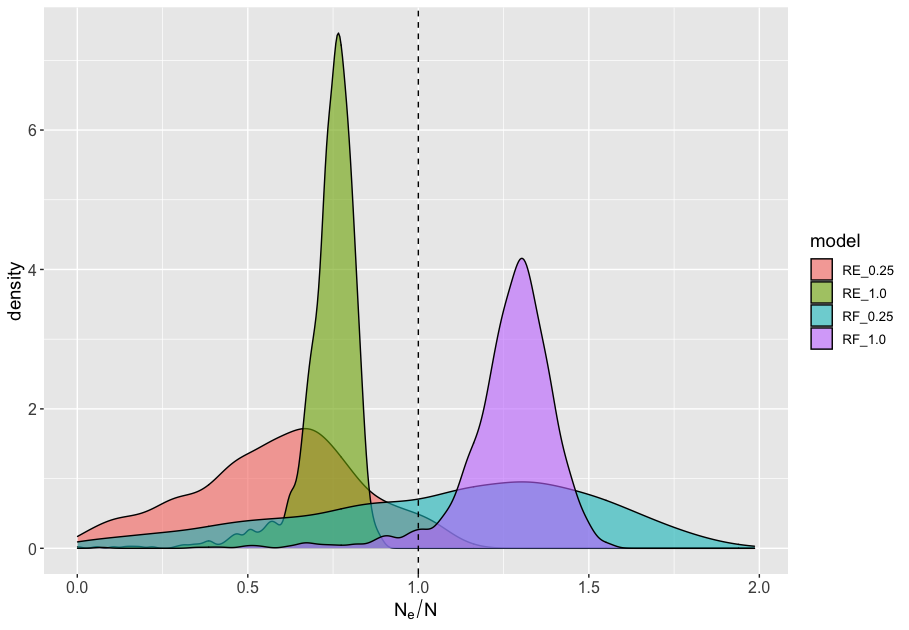
**Figure S3**. Density of *N_e_* / *N* following the split for each model. RE_0.25 = peripatric, 𝜎 = 0.25; RE_1.0 = peripatric, 𝜎 = 1.0; RF_0.25 = vicariant, 𝜎 = 0.25; RF_1.0 = vicariant, 𝜎 = 1.0. Dotted line represents when *N_e_* = *N*.

**Fig. S4.** Consensus trees produced by *BEAST2 with node calibrations signified by red stars. Node labels are posterior probabilities; branch labels are estimated lengths in substitutions, brackets are 95% HPD.


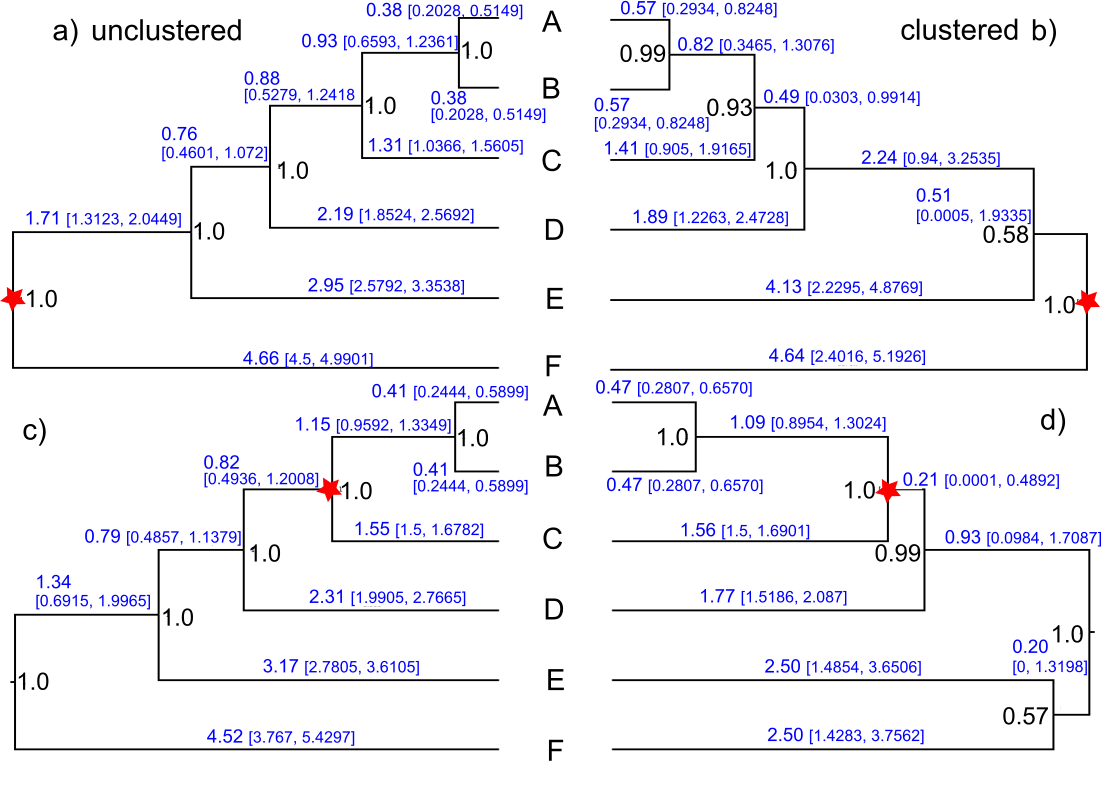


**Fig. S5**. Consensus trees from the SNAPP analysis. Node labels are estimated divergence times in numbers of substitutions per site; branch labels are estimated *θ*  values. Brackets beneath labels are the 95% HPD.


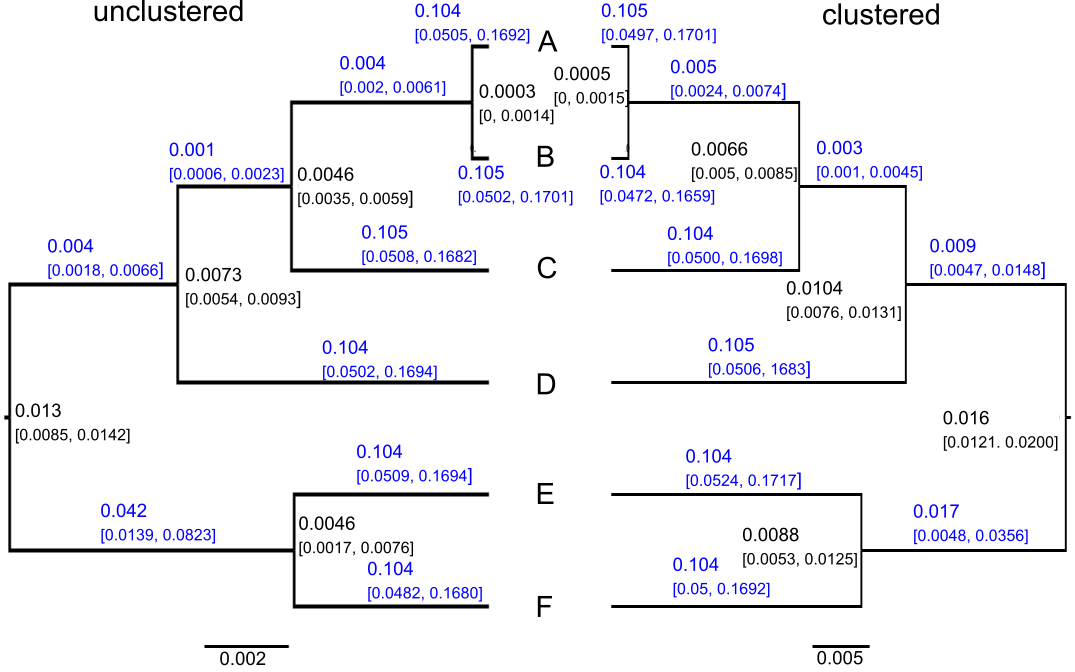

Supplement: supplementary material [file NIHMS1851696-supplement-supplementary_material.docx]
